# Drug susceptibility profiles of pulmonary *Mycobacterium tuberculosis* isolates from patients in informal urban settlements in Nairobi, Kenya

**DOI:** 10.1186/s12879-016-1920-5

**Published:** 2016-10-19

**Authors:** Glennah Kerubo, Evans Amukoye, Stefan Niemann, Samuel Kariuki

**Affiliations:** 1 Molecular and Experimental Mycobacteriology, Research Center Borstel, Borstel, Germany; 2 Centre for Microbiology Research, Kenya Medical Research Institute, Nairobi, Kenya; 3 Centre for Respiratory Disease Research, Kenya Medical Research Institute, Nairobi, Kenya; 4 German Centre for Infection Research (DZIF), partner site Borstel, Borstel, Germany

**Keywords:** Mycobacterium tuberculosis, Informal settlements, Drug resistance

## Abstract

**Background:**

Anti-tuberculosis drug resistance is an emerging health problem in Kenya and especially in slums. Slum environments create a conducive environment for the spread of tuberculosis (TB) due to high population density and lack of basic amenities such as decent housing, access to clean water, lack of drainage and basic sanitation. Furthermore, ineffective health services in crowded and poorer populations, poor patient compliance, a large pool of untreated cases, delayed diagnosis and inappropriate treatment regimens are likely to favour selection and spread of drug resistant *Mycobacterium tuberculosis (Mtb) strains* in such settings, however, precise data on this problem are only sparsely available. To address this question, this study aimed at determining drug resistance patterns of *Mtb* strains obtained from pulmonary TB patients who sought health care in randomly selected informal settings.

**Methods:**

This is a cross-sectional study conducted between September 2014 and March 2015, sputum samples were collected from 223 consenting adult patients and subjected to primary isolation and drug susceptibility testing. Socio-demographic data was collected and all data analysed using SPSS v20.

**Results:**

Drug susceptibility testing against first line drugs was successfully carried out on 184 isolates. Resistance to at-least one drug was observed in 33 % of the isolates. The highest prevalence of resistance to any drug was identified against isoniazid,(INH) (23.9 %) followed by Ethambutol (EMB) (13.6 %). The highest proportion of mono resistance was observed against INH, 25 (13.6 %). Multidrug resistance (MDR) was observed in 4.4 % of the new cases. There was no significant difference in the proportion of any resistance by sex, age or previous treatment.

**Conclusion:**

Levels of drug resistance have reached an alarming level in this population. Capacity of laboratories to conduct TB culture and DST should be strengthened in order to adequately manage TB patients and stop further creation and spread of MDR TB.

**Electronic supplementary material:**

The online version of this article (doi:10.1186/s12879-016-1920-5) contains supplementary material, which is available to authorized users.

## Background

Tuberculosis (TB) is an infectious disease that causes morbidity and mortality in the world, especially in poor resource settings and is often the first indicator of HIV infection [1, 2]. According to the world health organisation (WHO), there were an estimated 9.0 million incidence cases of TB globally [3]. More than half of these cases (56 %) were in the South-East Asia and Western Pacific Regions, while 29 % were in the African Region [3]. Global surveillance has shown that drug resistance towards TB threatens the progress made by TB control programs [4]. Inappropriate use of antibiotics in treatment of drug susceptible patients, sub-optimal treatment regimes and failure to complete treatment treatment in drug susceptible patients leads to drug resistance. Multidrug-resistant TB (MDR-TB), caused by *Mycobacterium tuberculosis* complex strains (MTBC) that are resistant to at least isoniazid and rifampicin, has become a menace in many parts of the world [5–7]. This has therefore resulted in high treatment failures and death rates due to the complexities in diagnosis and treatment [8, 9]. In 2013, WHO estimated that 480,000 people developed MDR-TB globally. Recent surveys have reported the emergence of untreatable form of TB, extensively drug resistant TB (XDR TB). These strains are resistant to isoniazid and rifampicin, in addition to any fluoroquinolone and at least one of three injectable drugs (amikacin, kanamycin, or capreomycin) [10, 11].

Kenya has a high TB burden and is ranked among the 22 high burden TB countries in the world, and has the fifth highest burden in Africa [3]. According to WHO, the MDR-TB prevalence in Kenya was 2.6 % in new cases and 13 % in retreatment cases [3]. TB is common in slums and resistant strains are also becoming more frequent in this population [12]. Especially, high population density, poor sanitation, poorly built housing with low lighting and air movement are likely to create a conducive environment for the spread of this pathogen in these slums. TB patients infected with drug resistant strains can easily infect upto 15 other individuals [12]. Nairobi houses more than 102 slums scattered in the expanse of the city [13]; more than half of the population now lives in slums which, however, cover just 5 % of the city land area [14]. Lack of employment opportunities in rural areas makes people to migrate to urban areas in search of job opportunities. Low income levels among many city migrants forces them to live in slum areas and hence complicates the situation further in these poor urban areas. In these informal settlements, people live in extreme poverty with most people making less than one US dollar a day [15].

Once introduced, drug resistant TB is likely to be spread in urban slums fostered by poor health services in overcrowded and poorer populations who do not stick to their prescriptions in combination with soaring rates of HIV. In addition, inconsistent, incorrect treatments being taken, delayed diagnosis or even unreliable drug supply contributes to drug resistance [16]. When TB control programs fail to adequately treat drug resistant forms of TB, then further resistance is acquired leading to the circulation of highly resistant of *M. tuberculosis* strains*. The effects of* generation of high-grade drug resistance due to incomplete treatment is not only grave for the patient, but for the community at large, since the transmission mechanism of drug resistant strains is similar to that of susceptible [17].

In turn, precise information on drug susceptibility patterns of circulating MTBC strains is an important factor of TB control and surveillance. Accurate and prompt detection of MDR strains is key to proper management of cases and is an indicator of the quality of TB control in the country. However, especially in slum setting, precise data on drug susceptibility patterns is only sparsely available.

To address this gap, this study aimed to establish the prevalence of drug resistance strains in selected urban slums of Nairobi and determine factors that may be important in driving the rise in cases. A cross sectional study was conducted in seven randomly selected health centres located in urban slums in Nairobi from September 2014 to May 2015. Samples obtained were subjected to primary isolation and drug susceptibility testing to the first line drugs.

## Methods

### Study setting and population

**Fig. 1:**
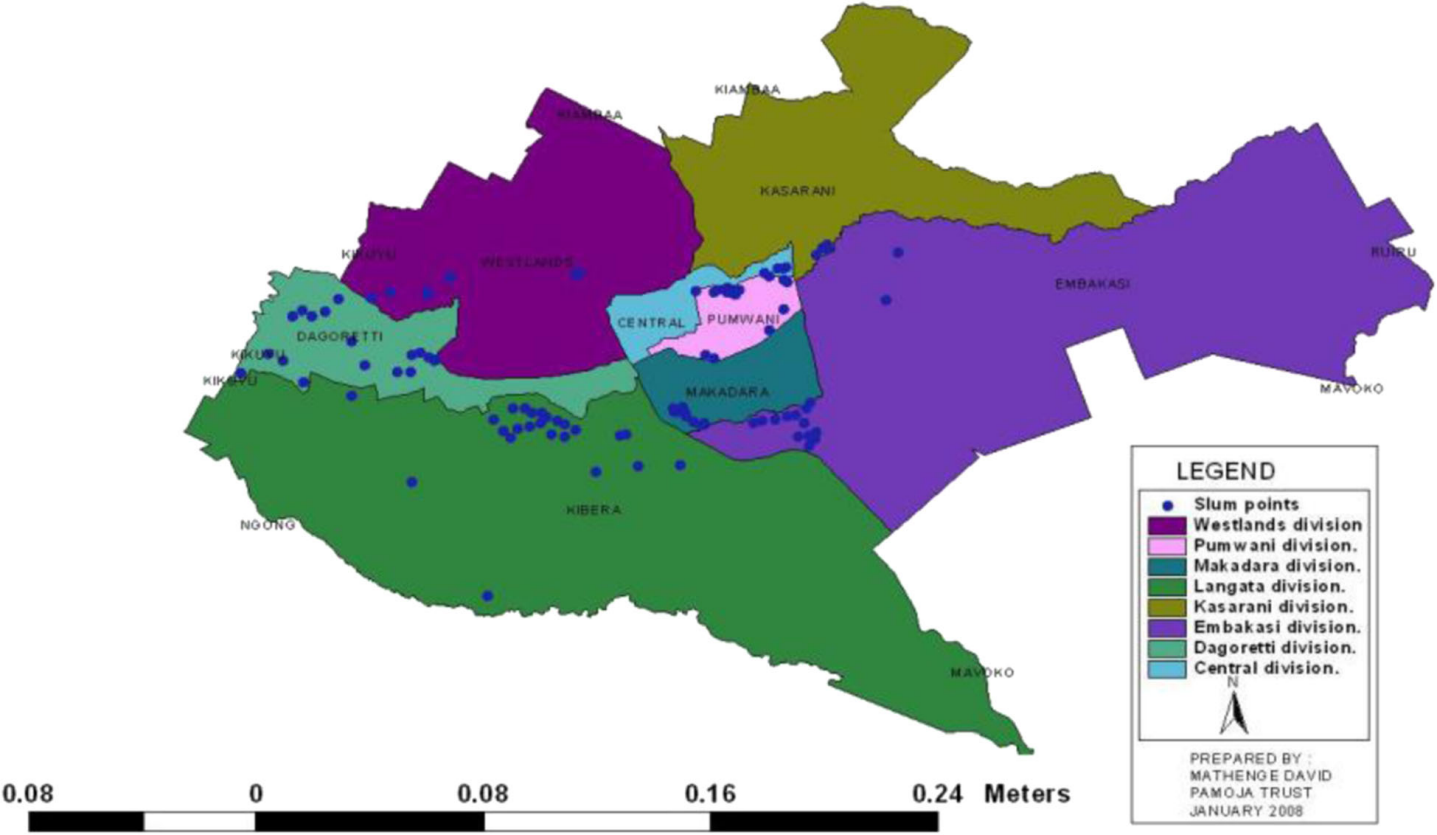
Map showing slums in Nairobi. Source: Source: http://sdinet.org/wp-content/uploads/2015/04/Nairobi_slum_inventory_jan_09.pdf. Nairobi province: distribution of infomal settlements

This cross sectional study was conducted in seven randomly selected health centres located in urban slums in Nairobi (Fig. 1) from September 2014 to May 2015. Majority of the patients seeking health care in these centres were from the slums. A significant proportion of the population in the slums lives below the poverty line and they try to sustain themselves through various means, including informal sector activities such as petty trade or casual labour Additional file 1.

Smear-positive cases of TB patients aged ≥18 years who consented to the study and living in slums were enrolled into the study. They completed a questionnaire containing demographic information, history of anti-TB treatment, history of contact with TB cases, alcohol usage and smoking history.

### Sputum collection and processing

Good quality spot and early morning sputum samples were collected in sterile 50 ml conical tubes (Corning®, MA, U.S.A) and then transported to the BSL 3 laboratory based in KEMRI in temperature controlled cool boxes. The samples were processed by the standard N-acetyl- L-cysteine (NALC)-NaOH method and concentrated at 3000 × g for 15 min. The sediment was reconstituted to 2.5 ml with phosphate buffer (pH 6.8) to make suspensions for the smears and cultures.

### Culture and identification

The sputum samples were cultured for the presence of *M. tuberculosis* on BACTEC MGIT 960 system (Becton Dickinson Microbiology systems, Sparks, MD, USA) according to the manufacturers’ recommendations [18]. The MGIT tubes (7 ml) were inoculated with the processed specimen and incubated at 37 °C in the BACTEC MGIT 960 machine. They were monitored automatically every 60 min for increase in fluorescence. The cultures were tested until positive for a maximum of 6 weeks. The isolates from MGIT 960 tubes were confirmed to be *M. tuberculosis* by microscopic observation for serpentine cord formation and inoculation onto blood agar plate to rule out contamination.

### Drug susceptibility testing

All *M. tuberculosis* strains were subjected to drug susceptibility testing (DST) to first line anti-tuberculosis drugs with an exception of PZA. DST using MGIT 960 was done following standard procedures according to the manufacturer’s recommendation [18]. Final drug concentrations were 1.0 μg/ml for Streptomycin (STR), 0.1 μg/ml for Isoniazid (INH), 1.0 μg/ml for Rifampicin (RIF) and 5.0 μg/ml for Ethambutol (EMB). The results were automatically interpreted by the BACTEC MGIT 960 instrument and reported as either susceptible or resistant.

### Quality control

The reference strain *M. tuberculosis* H37Rv (ATCC27294), which is susceptible to all standard anti-tuberculosis drugs, was used as susceptible control. All bacterial suspensions used for DST in MGIT 960 were checked for purity by culture on blood agar.

### Statistical analysis

Data was entered and analyzed by SPSS version 20.0 statistical software (SPSS Inc. Chicago). Descriptive analysis was done to depict the socio-demographic variables and proportions of drug resistant TB. Odds ratios (OR) and 95 % confidence intervals (95 % CI) were calculated to examine the association of risk factors and resistance. Bivariate analysis was used to evaluate the independent association between drug resistance and previous TB infection, previous TB treatment and contact with TB cases. Associations with *p*-values less than or equal to 0.05 were considered statistically significant.

## Results

A total of 223 sputum samples were collected from smear positive pulmonary tuberculosis (PTB) patients. Of these, 39 (17.5 %) were either culture negative or contaminated and thus excluded from further analysis. Among the 184 (82.5 %) included in this study, 160 (87 %) were new cases while 24 (13 %) were retreatment cases (Table 1). One hundred and twenty seven (69 %) were males, 110 (59.8 %) were married and 87 (47.3 %) had completed secondary school education with 39.7 and 12.5 % having acquired primary level and tertiary education respectively. A majority of the participants (75 %) were casual workers with 62 % of them living with less than five members in their households. Eighteen percent of them had a smoking history while 38 (20.7 %) consumed alcohol. The mean age of the patients was 32.09 (SD ± 7.69) ranging from 18 to 53 years. Table 1 describes the socio-demographic characteristics of the study population.

**Table 1:** Socio-demographic characteristics of participants

Characteristic	Male (*n* = 127)	Female (*n* = 57)	Total (*N* = 184)
Age			
18–23	9 (7.1 %)	12 (21.1 %)	21 (11.4 %)
24–29	39 (30.7 %)	17 (29.8 %)	56 (30.4 %)
30–35	32 (25.2 %)	17 (29.8 %)	49 (26.6 %)
36–41	30 (23.6 %)	9 (15.8 %)	39 (21.2 %)
42–47	11 (8.7 %)	2 (3.5 %)	13 (7.1 %
48+	6 (4.7 %)	0 (0.0 %)	6 (3.3 %)
Marital status			
Married	81 (63.8 %)	29 (50.9 %)	110 (59.8 %)
Single	44 (34.6 %)	26 (45.6 %)	70 (38.0 %)
Divorced	2 (1.6 %)	2 (3.5 %)	4 (2.2 %)
Education			
Never	0 (0.0 %)	1 (1.8 %)	1 (0.5 %)
Primary	48 (37.8 %)	25 (43.9 %)	73 (39.7 %)
Secondary	59 (46.5 %)	28 (49.1 %)	87 (47.3 %)
College	20 (15.7 %)	3 (5.3 %)	23 (12.5 %)
House type			
Homeless	2 (1.6 %)	0 (0.0 %)	2 (1.1 %)
Hut	17 (13.4 %)	11 (19.3 %)	28 (15.2 %)
Iron sheet	39 (30.7 %)	18 (31.6 %)	57 (31.0 %)
Stonewall	69 (54.3 %)	28 (49.1 %)	97 (52.7 %)
Source of Income			
Unemployed	9 (7.1 %)	5 (8.8 %)	14 (7.6 %)
Casual worker	97 (76.4 %)	41 (71.9 %)	138 (75.0 %)
Salaried	18 (14.2 %)	2 (3.5 %)	20 (10.9 %)
Dependant	3 (2.4 %)	9 (15.8 %)	12 (6.5 %)

DST was performed for all the 184 *Mycobacterium tuberculosis* isolates for the first line anti-TB drugs (INH, RIF, STR, and EMB) except for PZA. Prevalence of any resistance to at least one drug was 61 (33 %, 95 % CI 26.21–39.79). Any resistance to INH, STR, RIF and EMB was 44 (23.9 %), 13 (7.1 %), 12 (6.5 %) and 25 (13.6 %) respectively. The highest proportion of mono resistance was observed against INH, 25 (13.6 %), followed by EMB 11 (6 %), STR 4 (2.2 %) and RIF 1 (0.5 %). MDR was observed in 2 (8 %) of the retreatment cases and 7 (4.4 %) of the new cases. Only four (2.2 %) of all the isolates were resistant to all the drugs tested as shown in Table 2.

**Table 2:** Drug resistance patterns of *Mycobacterium tuberculosis* isolates

Category	New cases		Retreatment cases		All cases	
	*N* (%)	95 % CI	*N* (%)	95 % CI	*N* (%)	95 % CI
Total patients	*N* = 160		*N* = 24		*N* = 184	
Any Resistance	56 (35 %)	27.61–42.39	5 (20.8)	4.56–37.04	61 (33.1)	26.3–39.3
Any Resistance to:						
STR	10 (6.25)	2.5–10.0	3 (12.5)	0.73–25.73	13 (7.1)	3.39–10.81
INH	40 (25)	18.29–31.71	4 (20)	4.0–36.0	44 (23.9)	17.74–30.06
RIF	10 (6.25)	2.5–10.0	2 (8.3)	2.74–19.34	12 (6.5)	2.94–10.06
EMB	21 (13)	7.87–18.33	4 (16.7)	1.78–31.62	25 (13.6)	8.65–18.55
Mono Resistance to						
STR	4 (2.5)	0.08–4. 92	0 (0)	-	4 (2.2)	0.08–4.32
INH	25 (15.6)	9.98–21.22	0 (0)	-	25 (13.6)	8.65–18.55
RIF	1 (0.63)	0.6–1.86	0 (0)	-	1 (0.5)	0.52–1.52
EMB	10 (6.25)	2.5–10.0	1 (4.16)	3.83–12.15	11 (6.0)	2.57–9.43
INH + RIF Resistant (MDR)						
INH + RIF only	4 (2.5)	0.08–4.92	0 (0)	-	4 (2.2)	0.08–4.32
INH + RIF + EMB	1 (0.63)	0.6–1.86	0 (0)	-	1 (0.5)	0.52–1.52
INH + RIF + STR	0 (0)		0 (0)	-	0 (0)	-
INH + RIF + EMB + STR	2 (1.25)	0.47–2.97	2 (8.3)	2.74–19.34	4 (2.2)	0.08–4.32
Total MDR	7 (4.4)	1.22–7.58	2 (8.3)	2.74–19.34	9 (4.9)	1.78–8.02
INH + other Resistance						
INH + STR	3 (1.9)	0.22–4.02	1 (4.16)	3.83–12.15	4 (2.2)	0.08–4.32
INH + EMB	5 (3.1)	0.41–5.79	1 (4.16)	3.83–12.15	6 (3.3)	0.72–5.88
INH + STR + EMB	1 (0.63)	0.6–1.86	0 (0)	-	1 (0.5)	0.52–1.52
RIF + other Resistance						
RIF + EMB	1 (0.63)	0.6–1.86	0 (0)	-	1 (0.5)	0.52–1.52
RIF + STR + EMB	1 (0.63)	0.6–1.86	0 (0)	-	1 (0.5)	0.52–1.52

Bivariate analysis showed that there was no significant association of MDR with previous TB infection (*p* = 0.622, 95 % CI), previous treatment (*p* = 0.332, 95 % CI) or contact with TB cases (*p* = 0.693, 95 % CI) (Table 3).

**Table 3:** Bivariate analysis of various risk factors for MDR development

Category	OR	95 % CI		*P* value
		Lower	upper	
Previous TB treatment	1.987	0.388	10.18	0.332
Previous TB infection	1.714	0.337	8.728	0.622
Contact with TB case	1.535	0.368	6.401	0.693
Resistance to any one drug	3.365	2.68	4.27	0.001

## Discussion

Information on drug sensitivity patterns *MTBC* isolates against anti-TB agents is a crucial aspect in tuberculosis control and surveillance. Tuberculosis treatment for fully susceptible patients starts with a 4-drug regimen: isoniazid, rifampicin, pyrazinamide, and either ethambutol or streptomycin. After 2 months of therapy, pyrazinamide is stopped and the patient continues with isoniazid and rifampicin daily for 4 more months. Analysis of rates of resistance is helpful in understanding MDR TB trends and provides an indicator of the quality of TB control in the country. Furthermore, they help to identify hot spots of MDR TB e.g. in slum settings, thus, allowing targeted action to control further spread of MDR TB. Indeed, prompt and accurate drug resistance detection is important in the selection of the appropriate regimen to which the strain is susceptible to and in timely initiation of treatment. Early diagnosis also facilitates appropriate measures to prevent transmission.

In this study, resistance to one or more first line anti-TB drugs was 33 %. Compared to previous data, this is higher as reports from other studies conducted in Nairobi which found rates of 18.8 and 18.1 % [19, 20] and might indicate increasing resistance rates in the region. This is supported by a recent study by Ndungu et al. from a survey conducted in Nairobi who also reported resistance levels of around 30 % [21]. The differences in the drug resistance rates among the study areas could be due to sampling, inadequate diagnosis and treatment and poor patient compliance to treatment.

The findings of this study show the highest rate of mono resistance to be associated with INH (13.6 %). Previous studies conducted in Nairobi reported that the proportion of resistance to INH was within the range of 6.5–12.9 % [19–21]. Studies conducted in Ethiopia, Uganda and Zambia reported lower resistance rates of 9.5, 2.5 and 4.5 % respectively [22–24]. A higher INH resistance rate of 14.9 % was observed in Mozambique [25]. High INH rates could be associated with unfavourable treatment outcomes and its wide and longer use in treatment of TB.

In our study STR mono-resistance was 2.2 %. This rate is lower than similar studies conducted in Nairobi which reported rates of 5.1 and 5.2 % respectively [19, 21]. Higher STR rates of 14.8 and 26 % were reported in Benin and Ethiopia respectively [22, 26]. RIF mono-resistance was observed in only one isolate (0.5 %). Similar low resistance rates were reported in Nairobi and Uganda [20, 23]. The detected rate was however lower compared to other studies reported in Nairobi [19, 21, 22]. EMB monoresistance was 6 % in this study. Different resistance rates towards EMB have been reported in Nairobi, ranging from 2.6 to 8 % [19–21]. However, lower EMB rates of 0.3 and 0.9 % have been described in Ethiopia and Uganda respectively [22, 23].

The prevalence of MDR TB in this study was 4.9 %. Among new cases, the MDR TB rate was observed to be 4.4 and 8.3 % among retreatment cases. This proportion was however higher than the 4.3, 3.4 and 2.3 % reported in previous studies conducted in Nairobi on different populations [19–21]. Studies conducted in Ethiopia and Uganda and showed lower MDR TB rates of 1.4 and 2.3 % respectively [22, 23]. The findings of this study are however lower compared to the 7.7 % reported in Swaziland, 5.8 % in Mozambique and 5.2 % in Somalia [27–29]. Egypt also had a high MDR TB rate of 13.8 % [30]. This survey shows a probability that new TB cases were infected with a strain which is already drug resistant. There are several factors that could lead to high MDR TB in slums. Primary risk factors include poverty, malnutrition and overcrowding. A study by Muture et al. [31] found out that a majority of smear positive TB patients did not stay long enough in treatment to convert to smear negative. With the presence of defaulters combined with overcrowding and poor housing observed in the slums, the frequency and intensity of interpersonal contact is increased and this amplifies the risk of contagion droplet or airborne infection. The possible association between HIV infection in patients in slums and anti-TB drug resistance could also be a factor aiding the spread of TB. Mal-absorption of anti-TB drugs among HIV-positive patients, poor treatment adherence and lack of access to proper treatment could accelerate the spread of drug resistant TB [31]. Emphases on measures to effectively control infections within communities are necessary to improve on the large burden of disease. Intensified case finding and screening procedures will be beneficial to timely initiation of appropriate therapy and reduction of further transmission of TB. Rapid and accurate diagnosis of drug resistance should be accompanied by accessibility to second line drugs. Patients infected with drug resistant TB require supportive mechanisms to be able to comply to the lengthy treatment regimens.

One important limitation of this study is that HIV status patient data were not available for analysis, hence restricting our ability to derive concerete conclusions. HIV infection is the strongest risk factor for TB patients. In HIV infected individuals, TB is also the leading cause of death since it accelerates the course of HIV infection and increases viral load in some patients. HIV infection may also contribute to the increase in MDR TB among TB patients.

## Conclusion

This study demonstrates that the overall resistance to first line anti-TB drugs in Kenya is high. The highest mono resistance was detected against INH, also posing a potential risk to generate more MDR cases by ineffective treatment. The prevalence of MDR-TB is relatively high, signifying conditions favouring the spread of DR-TB are on the rise. Early case detection and treatment, better patient compliance, expanding diagnostic capacity for mycobacterial culture and drug susceptibility testing are vital steps to limit further spread of drug resistant TB strains in the slum setting. Prompt and accurate detection of drug resistance will be critical to timely initiation of treatment hence prevent further transmission and allows selection of a drug regimen to which the infecting strain is susceptible.

## Additional file

Additional file 1: Tuberculosis Health Assessment questionnaire. Socio- demographic data. Collects sociodemographic data such as age, sex, marital status, education level, type of housing, sources of income, number of people in a household and health behaviours. (DOC 33 kb)

## Abbreviations

DST: Drug susceptibility testing
EMB: Ethambutol
HIV: Human Immunodeficiency virus
INH: Isoniazid
KEMRI: Kenya Medical Research Institute
MDR TB: Multi drug resistant tuberculosis
Mtb: Mycobacterium tuberculosis
MTBC: Mycobacterium tuberculosis complex
OR: Odds ratio
PZA: Pyrazinamide
RIF: Rifampicin
SPSS: Statistical package for social scientists
STR: Streptomycin
TB: Tuberculosis
WHO: World Health Organisation
XDR TB: Extra drug resistant tuberculosis
