# Supplementary material for: Drug susceptibility profiles of pulmonary Mycobacterium tuberculosis isolates from patients in informal urban settlements in Nairobi, Kenya
Source: BMC Infect Dis. 2016 Oct 19;16:583. doi: 10.1186/s12879-016-1920-5 (PMC5070164; doi:10.1186/s12879-016-1920-5)
Supplement: Additional file 1: — Tuberculosis Health Assessment questionnaire. Socio- demographic data. Collects sociodemographic data such as age, sex, marital status, education level, type of housing, sources of income, number of people in a household and health behaviours. (DOC 33 kb) [file 12879_2016_1920_MOESM1_ESM.doc]

**Tuberculosis Health Assessment Questionnaire**

Patient number ------------------------------------------------------------------ Age --------------------------------- Sex ----------------------

Location----------------------------------------------------------------------------

1. What is your marital status?
2. Married
3. Single
4. Divorced/separated
5. What is the level of your education level?
6. Primary
7. Secondary
8. College/university
9. What kind of a house do you reside in?
10. Stone walled
11. Hut
12. Homeless
13. How many are you in your household?
14. >5 people
15. <5 people
16. Live alone
17. What is the source of your income?-------------------------------------------------------------------------------------------
18. Has anyone in your family suffered from tuberculosis before?
19. Yes
20. No.

If yes, when?----------------------------------------------

1. Have you been treated for TB before?
2. Yes
3. No.

If yes, for how long have you undergone treatment?---------------------------------

1. Have you been in contact with someone who has suffered or is suffering from TB?
2. Yes
3. No
4. Do you smoke?
5. Yes
6. No.

If yes, how many packets in a day?--------------------------------
